# Patient satisfaction with health care at a tertiary hospital in Northern Malawi: results from a triangulated cross-sectional study

**DOI:** 10.1186/s12913-022-08087-y

**Published:** 2022-05-24

**Authors:** Frank Watson Sinyiza, Paul Uchizi Kaseka, Master Rodgers Okapi Chisale, Chikondi Sharon Chimbatata, Balwani Chingatichifwe Mbakaya, Pocha Samuel Kamudumuli, Tsung-Shu Joseph Wu, Alfred Bornwell Kayira

**Affiliations:** 1 Mzuzu Central Hospital, Mzuzu City, Malawi; 2 Luke International, Mzuzu City, Malawi; 3 Faculty of Sciences, Technology and Innovations, Biological Sciences Department, Mzuzu University, Mzuzu City, Malawi; 4 Faculty of Applied Sciences, Department of Public Health, University of Livingstonia, Mzuzu City, Malawi; 5 Faculty of Health Sciences, Mzuzu University, Mzuzu City, Malawi; 6 University of Maryland Global Initiative Corporation, Lilongwe City, Malawi; 7 Pingtung Christian Hospital, Pingtung City, Taiwan

**Keywords:** Patient satisfaction, Client satisfaction, Quality of care, Health care

## Abstract

**Background:**

In 2016 the Malawi government embarked on several interrelated health sector reforms aimed at improving the quality of health services at all levels of care and attain Universal Health Coverage by 2030. Patient satisfaction with services is an important proxy measure of quality. We assessed patient satisfaction at a tertiary hospital in Northern Malawi to understand the current state.

**Methods:**

We conducted exit interviews with patients aged ≥ 18 years using a 28 statement interviewer administered questionnaire. Patients were asked to express their level of agreement to each statement on a five-point Likert scale – strongly disagree to strongly agree, corresponding to scores of 1 to 5. Overall patient satisfaction was calculated by summing up the scores and dividing the sum by the number of statements. Mean score > 3 constituted satisfaction while mean score ≤ 3 constituted dissatisfaction. A χ^2^ test was used to assess the association between overall patient satisfaction and demographic variables, visit type and clinic consulted at alpha 0.05. Patient self-rated satisfaction was determined from a single statement that asked patients to rate their satisfaction with services on a five-point Likert scale. We also asked patients to mention aspects of hospital care that they did not like. Responses were summarized into major issues which are presented according to frequencies.

**Results:**

Overall patient satisfaction was 8.4% (95% CI: 5.2 − 12.9%). Self-rated patient satisfaction was 8.9% (95% CI: 5.5 − 13.4%). There was no significant association between overall patient satisfaction and all predictor variables assessed. Patients raised six major issues that dampened their health care seeking experience, including health workers reporting late to work, doctors not listening to patients concerns and neither examining them properly nor explaining the diagnosis, shortage of medicines, diagnostics and medical equipment, unprofessional conduct of health workers, poor sanitation and cleanliness, and health worker behaviour of favouring relatives and friends over other patients.

**Conclusions:**

We found very low levels of patient satisfaction, suggesting that quality of services in the public health sector is still poor. It is, therefore, critical to accelerate and innovate the Ministry of Health’s quality improvement initiatives to attain Malawi’s health goals.

## Introduction

Malawi aspires to achieve Universal Health Coverage (UHC) by 2030 [1]. But a 2016 situation analysis of the health sector in Malawi identified low quality of care as a major setback to achieving UHC and improving population health outcomes [2]. Based on recommendations of this report the Malawi government has since 2016 been undertaking several health sector reforms to improve the quality of health services at all levels of care by improving and strengthening leadership and governance, human resource capacity, clinical practice, client safety, people-centered care and supply chain systems [1]. In 2018, the Ministry of Health and Population (MoHP) designated patient or client satisfaction with services as one of the main indicators for monitoring improvements in the quality of services in public health facilities in Malawi, and encourages the conduct of patient satisfaction surveys disaggregated by service type and facility type every two years [3]. The target is that by 2022 at least 80% of patients or clients seeking health care in public facilities should be satisfied with the health services provided [3].

Patient satisfaction, defined as the congruence between patient expectations of optimal care and the perception of the actual care received [4], is however not without limitations when used as an indicator for quality of care. According to the Donabedian quality of care model, health care quality encompasses the technical competencies of the providers as well as the interpersonal process through which that care is provided [5]. Technical quality of care is judged against the best in practice which is known or believed to produce the greatest improvements in health [5]. But Donabedian argues that due to limitation in medical knowledge most patients cannot competently assess the technical skills of their provider, and may therefore have low or no expectations on the technical quality of care [5]. As such, their satisfaction scores may only indicate the interpersonal skills of the provider and good health care outcomes. But even if the health care outcome is not good the quality of care given will still be judged as good if it conformed to best practice permitted by the science and technology of the day. Therefore satisfaction or dissatisfaction with care does not necessarily equate to receipt of good or bad quality of technical care.

Nonetheless, in recent decades, there has been a major shift in health care practice from the traditional way of defining quality of health care in terms of the technical standards to one that includes patient’s perception and judgement about the services received [6]. Patient perception about the quality of and trust in the health care services received has a huge bearing on their continued utilisation of health care and compliance with care regimens and suggested lifestyle modifications, which in turn affects treatment outcomes [7]. Donabedian, in his quality of care model, corroborates the importance of good interpersonal relationship between the provider and the patient because the interpersonal process serves as the vehicle by which technical care is implemented and on which its success depends [5]. Furthermore, beyond technical and interpersonal quality aspects of care, patient satisfaction has been reported to be influenced by availability and accessibility of health care providers, medicines and diagnostics; cost of services; and physical environment [8–10]. Studies have also demonstrated a direct connection between quality of services and patient satisfaction with services [11–13], making it an important indicator of health system performance improvement.

Four studies have previously assessed client or patient satisfaction in Malawi. All of them were done before the year 2016 and three of them reported satisfaction levels of more than 85% [14–16] which is higher than MoHP set target of 80% by the year 2022. The other study found that at least 75% of hospitalized stroke patients surveyed at discharge were satisfied with the care they received [17]. All four studies assessed satisfaction with either a specific service provided by a specific group of providers, one hospital unit or a specific group of patients, and mainly focused on the technical aspects of care. Perhaps this explains why MoHP set a target of 80% by the year 2022 knowing pretty well that studies done way earlier had demonstrated higher levels of client/patient satisfaction with the services. Motivated by these deficiencies and in responding to MoHP’s call for regular patient satisfaction surveys as a way of obtaining important feedback from clients and patients on the quality of services in public health facilities we assessed patient satisfaction with health care at a tertiary hospital in Northern Malawi.

## Methods

### Study design and setting

This was a descriptive cross-sectional study. It was carried out at Mzuzu Central Hospital (MCH) between January, 2021 and February, 2021 during the second surge of COVID-19 cases in the country. MCH is tertiary hospital located in Mzuzu City, Northern Malawi. It is a 410 bed capacity hospital and serves as the referral facility for six health districts that constitute the Northern Region and serving a population of 2,289,780 people [18]. Ideally the hospital provides specialist health services at the regional level. In practice, however, around 70% of the services it provides are either primary or secondary [19]. This is mainly due to unavailability of proper primary and secondary level health facilities in Mzuzu city and the surrounding areas and lack of a gate-keeping system [19]. Daily bed occupancy is at 410 and about 50 inpatients are discharged from hospital daily. Hospital records showed that the facility also treats about 500 patients daily on an outpatient basis.

### Data collection, management and analysis

We conducted exit interviews with patients aged 18 years and above shortly after having been discharged from hospital (for inpatients) or after they had completed consultation and received treatment. Interviewer administered structured questionnaire was used to collect data from patients. The questionnaire was adapted from the Patient Satisfaction Questionnaire (PSQ-III) and the Patient Satisfaction Questionnaire Short Form (PSQ-18) (an abbreviated version of PSQ-III), both of which are validated and reliable tools for assessing patient satisfaction with medical care [20, 21]. The adaption process involved rephrasing some statements to reflect the local context, dropping items that were not applicable locally, and substituting such items with those that were locally relevant. All new additions were based on literature. We engaged non-Mzuzu Central Hospital staff as interviewers to encourage free expression by patients. Interviewers had professional training in nursing and medicine. They were dressed in civilian clothes and interviews were conducted at a private place, away from identifiable hospital staff. Interviewers were trained in data collection tools and procedures before deployment.

The questionnaire consisted of 28 statements grouped into six domains of care: (1) Communication (4 statements), (2) Relational conduct (5 statements), (3) Technical skills/competence (5 statements), (4) Personal qualities/attributes (3 statements), (5) Availability and accessibility (6 statements), and (6) Physical environment (5 statements). Statements under communication domain solicited information from patients on whether health care providers provided adequate and patient tailored information on the investigations being done and eventual diagnosis, and adequately addressed patient concerns. Relation conduct domain comprised statements seeking information on whether patients were treated with respect by providers and were adequately involved in decision making. Statements under technical skills domain gathered information on whether providers demonstrated a masterly of their job. The personal attributes domain statements probed whether patients were treated courteously, and with privacy and empathy by providers. Statements under availability and accessibility domain solicited information on whether doctors were readily available and accessible to patients at the hospital and whether medicines, diagnostic services and functional medical equipment were also available. The physical environment domain asked patients to rate the adequacy, cleanliness and tidiness of sanitary facilities and hospital surroundings as well as the state of hospital infrastructure and room space.

Patients were asked to indicate their level of agreement to the statements on a five-point Likert scale: (1) Strongly disagree, (2) Disagree, (3) Not sure, (4) Agree, and (5) Strongly agree. The questionnaire contained a mix of statements expressing both positive and negative sentiments in a random order to minimize acquiescence bias. Together, the 28 statements provided a composite measure of satisfaction which we call Overall Patient Satisfaction. The questionnaire also contained one more question “*On the overall, how satisfied are you with the services you have received?*” with responses on a five-point Likert scale – very dissatisfied to very satisfied. The objective of this question was to solicit patients’ own subjective assessment of their health care seeking experience, herein referred to as Self-rated Satisfaction. The questionnaire further contained an open-ended question asking patients to mention any areas or aspects of care that needed improvement at the hospital. This question solicited inputs from patients so as to understand what constitutes quality health care from their perspective.

Data were entered in Microsoft excel 2016, cleaned and then imported into STATA V.13.0 (StataCorp) for analysis. But before any analysis could begin responses to all negatively framed statements were first re-coded so that all scores (1, 2, 3, 4, 5 corresponding to strongly disagree, disagree, not sure, agree and strongly agree) were in the same sense (i.e. the higher the score the higher the level of satisfaction). Overall patient satisfaction was calculated by summing up individual satisfaction scores across the six domains of care to get an overall score and then dividing this overall score by the total number of statements in the six domains. This calculation brought the overall scores back into the scale of 1 to 5. An overall mean score of more than 3 was treated as ‘Satisfied’ while an overall mean score of 3 or less was treated as ‘Unsatisfied’. This analysis was repeated for each domain to calculate domain specific overall satisfaction. Overall patient satisfaction was dichotomized because very few patients were satisfied with the care they received and splitting it further would have scattered the data even more, making it unlikely to observe any association between satisfaction and predictor variables.

For self-rated satisfaction responses very dissatisfied, dissatisfied and not sure constituted dissatisfaction whereas satisfied and very satisfied formed satisfaction. The response ‘Not sure’ was categorized on the dissatisfaction side because we felt that that was patients’ polite way of saying the services were not good. We believe if patients were happy with the services received they would not hesitate to say so. Patients’ responses to an open ended question were reviewed and summarized into major issues.

Descriptive statistics were performed to summarize patient characteristics. Overall patient satisfaction was the main outcome of analysis. A Chi square (χ^2^) test of independence was used to test the association between overall patient satisfaction and demographic variables, visit type and hospital clinic/department consulted. A χ^2^ test was performed for all cross tabulations where the sample size (n) was greater than the number of cells multiplied by 5 and where the expected value in 80% of the contingency cells was greater than 5 and no cell had the expected value of less than 3. Where this condition was not met a Fisher’s exact test was performed instead. A p-value of 0.05 or less was considered statistically significant. A binary logistic regression was not a good fit for the data at alpha 0.05 (i.e. Prob > chi2 was greater than 0.05) so we had to stick to the χ^2^.

## Results

A total of 225 patients were interviewed, representing 100% of the target sample size. Of these, 126 (56.0%) were female and the majority (38.7%) were in the 20–29 years age group. Half (50%) of the patients had completed secondary level education. The majority of patients resided in the Northern Region (77.8%), were treated as outpatients (58.7%) and were seen at the general outpatient department (44.4%) (Table 1).

**Table 1:** Characteristics of patients who participated in the study

Variable	Categories	n (%; 95% CI)
Sex	Male	99 (44.0)
	Female	126 (56.0)
Age (years)	< 20	15 (6.7)
	20–29	87 (38.7)
	30–39	56 (24.9)
	40–49	33 (14.7)
	50–59	15 (6.7)
	≥ 60	19 (8.4)
Education	None	8 (3.6)
	Primary	68 (30.2)
	Secondary	113 (50.2)
	Tertiary	36 (16.0)
Marital status	Married	150 (66.7)
	Single	56 (24.9)
	Divorced	9 (4.0)
	Widowed	9 (4.0)
	Other	1 (0.4)
Religion	Christian	217 (96.4)
	Muslim	7 (3.1)
	Other	1 (0.4)
Region of residence	Northern	175 (77.8)
	Central	24 (10.7)
	Southern	26 (11.6)
Visit type	Outpatient	132 (58.7)
	Inpatient	93 (41.3)
Department/clinic consulted	General OPD	100 (44.4)
	Obstetrics & Gynaecology	46 (20.4)
	Medical	37 (16.4)
	Surgical	22 (9.8)
	Dental	11 (4.9)
	Ophthalmology	5 (2.2)
	ART clinic	4 (1.8)

Overall patient satisfaction was 8.4% (95% CI: 5.2 − 12.9%), but ranged from as low as 4.9% for health worker attributes to as high as 27.1% for availability and accessibility of health workers and health services. Self-rated patient satisfaction was 8.9% (95% CI: 5.5 − 13.4%) (Table 2).

**Table 2:** Patient satisfaction with health care at tertiary hospital in Northern Malawi

Domain of care	Satisfaction with care	n (%)
Provider communication	Satisfied	40 (17.8)
	Unsatisfied	185 (82.2)
Rational conduct of the provider	Satisfied	20 (8.9)
	Unsatisfied	205 (91.1)
Provider technical competence	Satisfied	29 (12.9)
	Unsatisfied	196 (87.1)
Provider attributes	Satisfied	11 (4.9)
	Unsatisfied	214 (95.1)
Availability and accessibility	Satisfied	61 (27.1)
	Unsatisfied	164 (72.9)
Sanitation and cleanliness	Satisfied	17 (7.6)
	Unsatisfied	207 (92.40
Overall satisfaction	Satisfied	19 (8.4; 5.2–12.9)
	Unsatisfied	206 (91.6)
Self-rated satisfaction	Satisfied	20 (8.9; 5.5–13.4)
	Unsatisfied	205 (91.1)

A Chi square or Fishers’s exact test was used to explore associations between overall patient satisfaction and demographic characteristics of participants and other variables. None of the variables examined had a statistically significant association with overall patient satisfaction (Table 3).

**Table 3:** Association between patient characteristics and overall patient satisfaction

Variable		Overall Satisfaction		χ2 or Fisher’s exact (*p*-value)
		Satisfied *n* (%)	Unsatisfied *n* (%)	
Sex	Male	5 (5.1)	94 (94.9)	2.6 (0.11)
	Female	14 (11.1)	112 (88.9)	
Age (years)	< 20	0 (0.0)	15 (100)	… (0.14)
	20–29	12 (13.8)	75 (86.2)	
	30–39	2 (3.6)	54 (96.4)	
	40–49	4 (12.1)	29 (87.9)	
	50–59	1 (6.7)	14 (93.3)	
	≥ 60	0 (0.0)	19 (100)	
Education	None	0 (0.0)	8 (100)	… (0.97)
	Primary	5 (7.4)	63 (92.6)	
	Secondary	11 (9.7)	102 (90.3)	
	Tertiary	3 (8.3)	33 (91.7)	
Marital status	Married	11 (7.3)	139 (92.7)	… (0.53)
	Single	7 (12.5)	49 (87.5)	
	Divorced	1 (11.1)	8 (88.9)	
	Widowed	0 (0.0)	9 (100)	
	Other	0 (0.0)	1 (100)	
Religion	Christian	19 (8.8)	198 (91.2)	… (1.00)
	Muslim	0 (0.0)	7 (100)	
	Other	0 (0.0)	1 (100)	
Region	Northern	15 (8.6)	160 (91.4)	… (1.00)
	Central	2 (8.3)	22 (91.7)	
	Southern	2 (7.7)	24 (92.3)	
Visit type	Outpatient	14 (10.6)	118 (89.4)	1.9 (0.17)
	Inpatient	5 (5.4)	88 (94.6)	
Department consulted	General OPD	13 (13.0)	87 (87.0)	… (0.26)
	Obs & Gynae	4 (8.7)	42 991.3)	
	Medical	0 (0.0)	37 (100)	
	Surgical	1 (4.5)	21 (95.5)	
	Dental	1 (9.1)	10 (90.9)	
	Eye	0 (0.0)	5 (100)	
	ART clinic	0 (0.0)	4 (100)	

The top six areas of improvement cited by patients are that health workers should report to work on time at 29.8% followed by a plea that doctors should listen to patients’ concerns, examine them thoroughly and explain their findings and diagnosis, including the reasons for doing blood tests and other examinations at 17.8% (Table 4). *“Doctors should come to work on time and examine patients properly”*, said a 27 year-old male when asked what he thought could have been done differently to improve his experience at the hospital. While a 35 year-old female had this to say *“Doctors should pay attention to patients and examine them properly based on their complaints*”.

Third was an observation that the hospital should improve its stocks of essential medicines, diagnostics and medical furniture at 14.7%, which was followed by an earnest call that health workers must conduct themselves professionally at 12.9% (Table 4). *“Doctors should minimize chatting with colleagues and on their phones when attending to us”* said a 21 year-old woman when asked what should improve at the hospital to make her experience better. Another 20 year-old female responded “*Stock enough drugs in the pharmacy and provide more chairs on the outpatient queues so that we can observe social distance during this era of COVID-19”*.

On fifth position, there was a call from 6.7% of patients that hospital management has to improve cleanliness in the hospital’s sanitary facilities and regularly maintain the physical infrastructure. Finally at number six, 4.0% of patients bemoaned the behaviour of some health workers who favour or prioritise their relatives and friends over other patients and pleaded that heath workers should change this discriminatory behaviour (Table 4). When asked what she thought should have been done differently in order to improve her experience at the hospital a 60 year old female said *“improve sanitation in the toilets”* while 63 year old woman said *“stop prioritizing relatives and friends of health workers and treat us all equally”.*

Seventy two patients (32%) contradicted their initial responses and said they were satisfied with the services they received when  prompted to suggest what could be improved at the hospital (Table 4). When asked to mention areas that needed improvement at the hospital so as to make their experience better next time they come to seek care a 42 year old male said *“I’m satisfied with the services”*. An 18 year old female said *“The hospital should keep up the good work it is doing”* while a 36 year old female said *“There’s improvement on abuse of patients and that should continue”.*

**Table 4:** Patients identified areas of improvement in health service delivery at the hospital

Area of improvement	Number of patients who mentioned it n (%)
Timely reporting to work	67 (29.8)
Doctors should listen to patients concerns, examine them properly and explain the diagnosis	40 (17.8)
Unavailability/frequent stock outs of essential health commodities (medicines, diagnostics, medical equipment/furniture)	33 (14.7)
Professional conduct of health workers	29 (12.9)
Sanitation and cleanliness	15 (6.7)
Health workers should not favour/priotise their relatives over other patients	9 (4.0)
Provide adequate physical space to be able to observe social distancing in the wake of COVID-19	6 (2.7)
Reduce by-pass fees	6 (2.7)
Continuity of care – maintain same doctors during the next visits	3 (1.3)
Technical competence of health workers	3 (1.3)
Unity among health workers	2 (0.9)
Health workers should stop soliciting/taking bribes from patients	2 (0.9)
Availability and accessibility of doctors	1 (0.4)
Supervision and control of students on clinical placements	1 (0.40
Satisfied with the services	72 (32.0%)^a^

^a^Of the 72 patients that said they were satisfied with the services they received 71 (98.6%) of them were not satisfied with the care they received in our objectively measured overall satisfaction, and all of them (100%) reported not being satisfied in their self−rated overall satisfaction. Forty two (58.3%) of them had completed at least secondary school education

## Discussion

 We assessed patient satisfaction in six domains of care (communication, rational conduct, technical competence, personal qualities, availability and accessibility, and physical environment) and calculated an overall measure of patient satisfaction. We also report patient self-rated satisfaction with the services they received and patient suggested areas of improvement for better service delivery at the hospital. To our knowledge this is the first study in Malawi to have taken a multi-pronged approach to assessing patient satisfaction, and to have assessed satisfaction holistically and not focussing on a specific service or hospital department.

Both overall patient satisfaction self-rated satisfaction were low (8.4% and 8.9% respectively), suggesting that the quality of services in public hospitals is still not satisfactory. If this study had included family members of patients who died in hospital the service ratings would have been even poorer considering the fact that patients who survive often tend to rate services as satisfactory [22]. It is encouraging to note, however, that our measured overall satisfaction was not different from patient self-rated satisfaction, giving confidence in the tool that we used to objectively assess patient satisfaction. Therefore, instituting improvements in the domains of care that we assessed may lead to increased satisfaction with care among patients.

Previous studies reported high levels of satisfaction with health care services in Malawi. In a study investigating client satisfaction with cervical cancer screening all women (100%) reported being satisfied with the services, with 68.3% reporting being very satisfied [14]. Creanga and colleagues found patient satisfaction levels of more than 85% with perinatal care [15]. 97% (97%) of women were satisfied with reproductive health services at Gogo Chatinkha Maternity Unit in Blantyre, Malawi [16] while more than 75% of stroke patients were said to be satisfied by the care they received in four tertiary hospitals in Malawi [17]. All of the above studies have fundamental differences from our study. While we attempted to assess the hospital as a system, encompassing as many dimensions of care that might lead to patient satisfaction (or otherwise) as possible, they focused on a specific service provided by specific staff in a particular unit or department of the hospital. Taking such a narrow approach one is likely to find higher levels of satisfaction. In Nigeria and Uganda studies that assessed a particular service or one aspect of care provided by the hospital or clinic reported higher levels of satisfaction (91.6% and 93.8% respectively) [23, 24].

The hospital, however, is a much broader sand complex system. In navigating such a system patients may encounter several frustrations along the way, including having to interact with multiple providers with varying technical competencies and personal manners, and from different professional backgrounds. In resource constrained countries like Malawi patients are also faced with limited access to the doctor, frequent stock outs of essential medicines and limited diagnostics services. Studying patient experiences with the health care system from such a broader perspective one may find lower levels of satisfaction. In Ethiopia and Uganda, studies that took a similar approach to our own and measured patient satisfaction in a similar manner found lower levels of satisfaction with nursing care among hospitalized patients (40.7% and 49.2%) [25, 26], inpatient services (46.2%) [27] and outpatient services (50.0%) [28]. Even though our results are still far lower than these the trend is apparent, and the observed discrepancies could be due differences in study sites. We are, therefore, of the view that when assessing patient satisfaction with hospital care taking a holistic approach is the best way to draw out true hospital ratings from the people it endeavors to serve better.

Further, this study was conducted in the midst of the COVID-19 pandemic. COVID-19 has had significant impact on the delivery of other essential health services in Sub-Saharan Africa, including Malawi. It led to shortage of human and material resources due to staff and money being redirected to tackle the epidemic [29, 30]. COVID-19-related stressors such as physical exhaustion, alarming deaths of COVID-19 patients and the fear of contracting infection and subsequently passing it to family members took a huge toll on mental health of health workers [31, 32], which in turn may have affected how providers related with patients. Globally, COVID-19 lockdowns disrupted supply chains and lead to acute shortage of medicines and other essential health commodities in Malawi [33, 34]. In addition, the global scramble for essential health commodities such as masks and other protective equipment (PPE) led to severe shortages of these items in third world countries like Malawi [35]. Without appropriate and adequate PPE it was difficult for health workers to maintain good provider-patient interactions and discharge their duties comfortably. A combination of these factors may have plummeted health care provider and hospital ratings in the eyes of the patient.

We examined the association between overall patient satisfaction and independent variables listed in Table 1 using a Chi square or Fishers’s exact test, as appropriate. Initially, the plan was to fit a binary logistic regression but when we attempted to fit such a model with either of the variables individually or together the model itself was insignificant at alpha 0.05. As such we had to explore the association using basic statistical tests (Chi square or Fisher’s exact) which too did not reveal any association at 5% level of significance. Maseko et al. found no association between client satisfaction with cervical cancer screening and age, education level or marital status [36] while Nabbuye-Sekandi and colleagues reported higher levels of satisfaction among clients with primary or secondary education compared with those that had no formal education [28]. They also found greater levels of satisfaction among clients who attended certain specialized clinics (HIV treatment and research clinic) than among those who attended general outpatient clinics [28]. Sharew et al. reported the opposite of what Nabbuye-Sekandi et al. had reported. In their study they found that patients with at least primary education were 80% less satisfied compared with those without any formal education [26]. So, failure by our study to find any significant associations between satisfaction and demographic variables, visit type and department or clinic consulted could mean that indeed there is no association, or simply a failure by our study to detect these associations owing to few events (only 8.4% of patients were satisfied and therefore could not achieve adequate distribution for optimal comparison).

Patients raised various issues that dampened their health care seeking expereince at the hospital. Top on the list were health workers reporting late to work, that doctors do not listen to patients’ concerns and that they do not take time to examine patients thoroughly and explain the findings, shortage of medicines and diagnostics, and unprofessional conduct of health workers. Five of the top six items raised by patients were already included in the questionnaire we used to objectively assess patient satisfaction, giving reassuarance that the tool we used touched on issues that patients considered important. A small proportion of patients also raised some important issues that the hospital should consider improving if it is to appeal to its clientele. Concerns that health workers are favouring or prioritizing their relatives and friends over ordinary patients by aiding them to skip the queue, the revelation that some health workers are soliciting bribes from patients, and the need for adequate physical space so that patients can observe social distance while waiting on the queue during the COVID-19 pandemic must be seriously looked into. None of the issues raised were related to the technical aspects of quality of care. Nonetheless, these are the things that patients are able to observe and upon which they base their evaluation of the performance of the hospital. Therefore, while aiming to improve the technical quality of care particular attention must be paid to the nontechnical aspects of it as well.

When asked to mention areas that the hospital should improve to meet their expectations a substantial proportion of patients (32%) had nothing specific to point a finger at other than to contradict their earlier statements and say they were satisfied with the care they had received. Of these, 98.6% were not satisfied with the care they received by our measured overall satisfaction, and all of them (100%) reported not being satisfied in their self-rated satisfaction. Forty-two (58.3%) of them had completed at least secondary education. This contradictory result is interesting. We suspect that despite many of them having good education they still lacked knowledge on their rights with regard to health care, and therefore had no expectations of the quality of services they ought to receive. Without expectations it is difficult to judge the actual care received, and hence unable to point out a single thing that was not right in the services they received.

## Conclusions

Patient satisfaction was very low, suggesting that patients were not happy with the quality of services they received. This is a strong message to policy makers and health managers to improve the quality of services and patient experience in public hospitals. To stay true to its commitment of improving population health outcomes and achieve UHC by 2030 through provision of quality services the Malawi government has to step up and accelerate current initiatives meant to improve quality of services or innovate its quality improvement approaches. Furthermore, the Malawi health system has to get better prepared for future pandemics because these tend to reverse the gains made in previous years. In addition, the Malawi government and development partners should consider sensitizing citizens on their rights and responsibilities enshrined in the Malawi service charter on patients’ and health service providers’ rights and responsibilities so that they know what to expect from and what is expected of them during a health care seeking encounter. Until there is congruence between expectations of ideal care and the actual care received it will be difficult for patients to rate the services as satisfactory or not. So even if the Malawi government is to make investments to improve quality of care and patient experience in its facilities future patient satisfaction surveys may still fail to detect changes in levels of satisfaction as patients may not be able to distinguish between optimal and suboptimal care.

## Data Availability

The datasets used and/or analysed during the current study are available from the corresponding author on reasonable request. Email: kayiraalfred@gmail.com.

## Abbreviations

MOHP: Ministry of Health and Population
UHC: Universal Health Coverage
PPE: Personal Protective Equipment
